# A School Nurse Competency Framework for Continuing Education

**DOI:** 10.3390/healthcare8030246

**Published:** 2020-07-30

**Authors:** Eun Mi Shin, Young Sook Roh

**Affiliations:** 1 Hwagok Health Management High School, Seoul 07638, Korea; embolism1996@naver.com; 2 Red Cross College of Nursing, Chung-Ang University, 84 Heukseok-ro Dongjak-gu, Seoul 06974, Korea

**Keywords:** competency, continuing education, focus group interview, school nurse

## Abstract

Background: This study develops a school nurse competency framework for continuing education based on focus group interviews and a literature review. Methods: This study uses a qualitative content analysis with 12 school nurses. Six school nurses verify the content validity for the competency framework for continuing education using the content validity index. Results: School nurse competencies are defined as the knowledge, skills, and attitudes required of school nurses to provide safe school nursing. Six core competencies are identified. These include the ability to (1) provide patient-centered care; (2) communicate and collaborate with students, teaching staff, and community resources; (3) think critically for evidence-based practice; (4) implement school health services and programs; (5) integrate legal and ethical nursing practice, and (6) conduct health education. Conclusion: It is necessary to develop and implement continuing education programs for school nurses based on the training needs and competency indicators identified in this study.

## 1. Introduction

The school nurses play an essential role in coping with a population with a variety of health issues in a school setting [1]. Some surveys identified an average school nurse-to-student ratio as high as 1:1086 in the United States [2], and 1:1000 in the Republic of Korea, respectively [3]. However, the performance of health-related work in school nursing depends largely on the individual competencies of school nurses, precipitating a demand for a systematic education approach to strengthen their competency. The roles or competencies required for school nurses are various and inconsistent [4,5], highlighting the need for the development and implementation of a population-focused definition of roles and competencies. School nurses in the United States are charged with primary, secondary, and tertiary prevention modalities [6]. Through these school nurse-led practices, school nurses contribute to the health and well-being of the students [7] as well as the students’ academic success [8].

School nurses with optimal competency can contribute to student health and success, so they require a competency-based continuing education program. Competency-based continuing education is defined as an educational experience to facilitate the application of learning in its appropriate context-knowledge, skill, and/or practice and decrease the professional practice gap [9]. Some studies have identified the roles, standards, or competencies of school nurses. The main roles of a school nurse include various activities to improve the health of students, families, and the community, such as offering health education, clinics, referrals, immunization sessions, health screening, and care planning [7]. The Council on School Health [4] explains the role of the school nurse as providing both individual and population health services, chronic disease management, emergency preparedness, behavioral health assessment, ongoing health education, and extensive case management to address and coordinate the healthcare needs of children and adolescents. The National Association of School Nurses (NASN) Framework for 21st Century School Nursing Practice includes four domains: (1) care coordination (2) leadership (3) quality improvement, and (4) community/public health [5,10].

School nurses in Korea are obliged to complete job training as “a teacher” and continuing education as “a registered nurse” to maintain their jobs. Continuing education is required for school nurses to acquire the knowledge, skills, and attitudes needed to provide high-quality school nursing to the target population. Therefore, it is necessary to systematically develop a continuing education program considering the school nurses’ competencies and careers. There is a high demand for continuing education based on a theoretical framework and evidence, but the related research on school nurses is still insufficient. The framework provides a basis for developing both a continuing education curriculum for the professional development of school nurses and standards of school nursing practice. 

Although school nurses play a pivotal role in school health, there is a lack of qualitative studies identifying the training needs of school nurses in competency-based continuing education programs. Qualitative methods of inquiry enable comprehensive consideration of the perception of the school nurse [7]. Some studies reported on the role, standards, or competencies required of school nurses, but they have been inconsistent in defining core competencies of school nurses. Thus, it is necessary to identify the key school nurse competencies based on the perception of school nurses and a literature review.

### Purpose

This study aims to develop a school nurse competency framework for a continuing education curriculum based on focus group interviews (FGIs) and a literature review. The specific purposes are to explore the school nurses’ perception of continuing education through the FGIs and develop a school nurse competency framework for continuing education.

## 2. Materials and Methods

### 2.1. Phase I: Needs Assessment of School Nurses Using Focus Group Interviews

#### 2.1.1. Design

The present study employed a qualitative exploratory approach with FGIs to gain an understanding of the school nurses’ perceptions. The present study conducted and reported the FGIs based on the consolidated criteria for reporting qualitative research [11].

#### 2.1.2. Participants

The appropriate sample size for FGI is 4~12 people [11]. The researcher approached and recruited the 12 study participants via telephone from 11 school districts in Seoul using a purposive sampling method. Taking into consideration that X city has the highest placement rate of school nurses in the Republic of Korea, the researcher selected the school nurses in this area as participants. The researcher recruited participants with at least 3 years of school nurse experience and who had completed the first-grade qualification training for school nurses. All 12 invited school nurses who were working at middle (n = (6) and high schools (n = (6) completed the interviews. All 12 participants were female, with an average age of 51.8 years. The mean number of years working as a school nurse was 23.7.

#### 2.1.3. Data Collection

Data collection occurred at a conference room at the College of Nursing, Seoul, Republic of Korea. The author held focus group interviewswith six school nurses from middle schools on 26 October 2015, and an FGI with six school nurses in high schools on 27 October 2015. The interviews lasted two hours each.

An interview guide with clear, structured, open questions that elicited the participants’ experience was developed and pilot-tested by the author. The two topics of this study were “experiences in current continuing education” and “preferred themes for competency-based continuing education.” Prior to starting the FGIs, we provided light meals and refreshments to encourage participants to greet each other and chat in a relaxed setting. The opening questions included questions that introduced each other. Through the introductory questions, participants were encouraged to think about the topic and relate it to themselves through open questions about their school’s emergency and first aid experience. The transition question posed the idea of continuing education for school nurses and led to a full-scale discussion process closer to the subject of this study. The key questions were pertinent to focusing the nurses on the study themes: direct and indirect experience in continuing education, how to complete continuing education, how helpful was the continuing education they have participated in so far in their job and why, and desired themes for continuing education. The final questions allowed participants to reflect on previous comments.

The author audio-recorded the interviews to collect the data with the consent of all participants and annotated field notes during the interviews. The author conducted the semi-structured FGIs, and one research assistant performed the recording. A research assistant who participated in the FGIs transcribed the recorded interviews on the interview days. The author listened to the recordings repeatedly and checked the transcripts. Regarding cases requiring additional confirmation, the author contacted the research participants individually. Data saturation was reached by identifying that no new themes emerged from the focus groups [12].

#### 2.1.4. Ethical Considerations

The institutional review board of the Chung-Ang university (Approval No.: 1041078-201510-HRSB-172-01) approved this study. The researcher informed participants that they were free to refuse to participate or withdraw from participation in the study at any time without penalty. We obtained written informed consent from each participant after explaining the anonymity and confidentiality of data.

#### 2.1.5. Data Analysis

This study employed an inductive content analysis approach. Data included transcripts of recordings, field notes, oral summaries (notes) in the focus group, and debriefing notes immediately after the focus group. Concerning data analysis, the researcher repeatedly read the transcript, analyzed and interpreted the contents without using data analysis software. The research assistant uninvolved in the focus group interviews also reviewed one copy of the summary to verify the validity of the analysis content. One professor of nursing with qualitative research experience confirmed and corrected the relevance of the content.

### 2.2. Phase II: Developing a School Nurse Competency Framework for Continuing Education 

**Figure 1:**
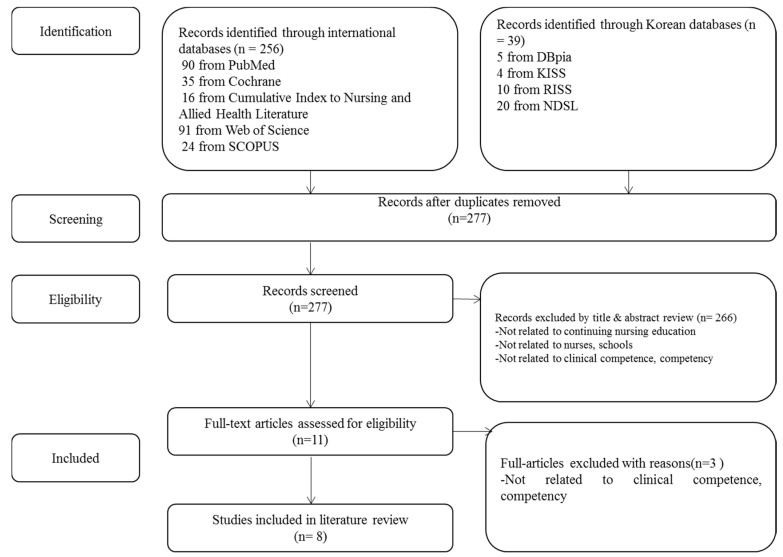
Flow chart of the study selection process.

Regarding a narrative literature review, the researchers searched published articles using a Medical Subject Headings term (clinical competency, continuing nursing education, nurses, schools, school nursing) in the international (PubMed, Cochrane, Cumulative Index to Nursing and Allied Health Literature [EBSCO], Web of Science, and SCOPUS) and Korean databases (DBpia, KISS, RISS, and NDSL) published from 1 January 2005, to 31 May 2015. During this study, the literature was selected according to the inclusion and exclusion criteria, and the selection process of the literature was described step-by-step using the Preferred Reporting Items for Systematic Review and Meta-Analysis (PRISMA) flow chart (Figure 1).

The present study defines school nurse competencies as the knowledge, skills, and attitudes required of school nurses to provide safe school healthcare. The author proposed the initial draft regarding school nurse competency based on the literature review and Dr. Benner’s novice-to-expert model [13], including six domains and 27 competency items. Content validity was verified by a group of six experts (one school nurse with at least 6 years of experience as a continuing education instructor, one FGI participant, two school nurses currently in charge of consulting, and two supervisors of health at school districts in Seoul city). Six school nurses verified the content validity of the school nurse competency items using a 4-point Likert scale (1 = very inappropriate; 4 = very appropriate). The author finalized all competency items with acceptable item-level content validity indices of at least 0.80 to develop a school nurse competency framework for continuing education.

## 3. Results

### 3.1. Phase I: Needs Assessment of School Nurses 

Using focus group interviews, the authors separated the perceptions of participants on current continuing education into three areas, seven categories, and 25 subcategories: (1) continuing education that is difficult to apply (required continuing education, continuing education that is difficult to apply on the school site, continuing education behind reality); (2) continuing education to strengthen competency (knowledge acquisition, job promotion), and (3) continuing education for professional development (professionalism, personal healing).

#### 3.1.1. Continuing Education That Is Difficult to Apply

Participants, as teachers and nurses, were required to receive continuing education. However, because the educational content did not reflect the reality well, the participants considered it out of date and difficult to apply to the school setting. Participants complained about the content of continuing education, which was not practical in school settings. The following is a quote by Participant 2:

“As a school nurse, I must complete 60 hours or more in accordance with each school standard as a teacher performance evaluation standard. However, it is difficult to apply to the school setting because the educational content is behind the reality.”

#### 3.1.2. Continuing Education to Strengthen Competency

To contrast, participants were satisfied with the continuing education on the latest school health issues with learner-centered educational strategies, such as the use of videos and task-based practice to acquire competency. Participant 5 said, “It was different from the education that I received when training in cardiopulmonary resuscitation at an external institution, and it was good to practice in person.”

#### 3.1.3. Continuing Education for Professional Development

Participants said that school nurses must have competency-based continuing education to fill the gaps between their actual competency and optimal competency: “Continuing education that reflects the changing healthcare environment and the role of health teachers is needed. In addition, differentiated continuing education is needed according to the expertise and experience of school nurses” (Participant 7).

#### 3.1.4. Preferred Themes for Continuing Education

Table 1 shows the school nurses’ preferred themes for continuing education composed of three areas, six categories, and 19 subcategories. Themes include training regarding the assessment and treatment of common, severe, and rare diseases; dispute resolution; managing school violence and managing the school health room. Considering these themes, their most preferred was basic life support, followed by first aid, safety, and accidents.

### 3.2. Phase II: Developing a School Nurse Competency Framework for Continuing Education 

Regarding the competency and continuing education in school nursing, 295 documents (international 256, Korean 39) were searched in the databases. After removing 18 documents that were duplicated, the researcher reviewed 277, based on the title and abstract of the study. The researcher reviewed the title first, reviewed the abstracts for ambiguous papers, and selected 11 articles, except for 266 items that did not meet the criteria. Consequently, after reviewing 11 papers according to the same criteria and process, mainly on the original text, eight items, except for three, were finally selected (Figure 1). Results from a narrative literature review identified six school nurse competency attributes: (1) patient-centered care [6,14,15,16,17]; (2) communication and collaboration [15,16,17,18]; (3) evidence-based practice [16]; (4) school health services and programs [6,14,16,17]; (5) legal and ethical nursing practice [19], and (6) health education [16,17,18]. One study proposed a framework using principles of Quality and Safety Education for Nurses (QSEN) in school nurse continuing education related to child neurology. The framework has six QSEN competency domains: (1) patient-centered care; (2) teamwork and collaboration; (3) evidence-based practice; (4) quality improvement; (5) safety, and (6) informatics [1].

**Figure 2:**
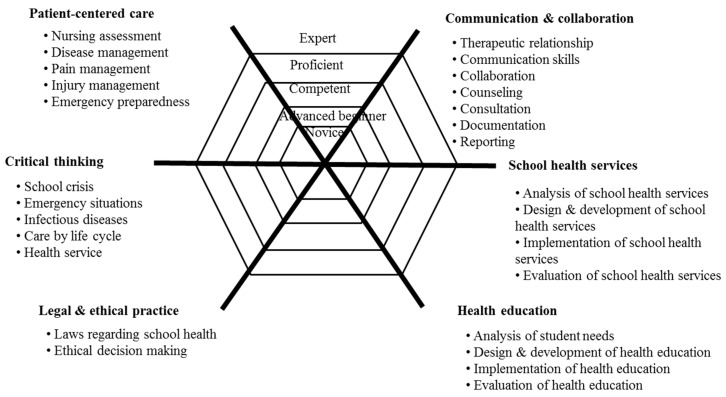
A school nurse competency framework for continuing education.

The final framework was developed based on the focus group interviews and literature review (Figure 2). It shows the six core competencies of school nurses and the key roles of each core competency. The six core competencies include the ability to (1) provide patient-centered care through the integration of knowledge and skills; (2) communicate and collaborate with students, teaching staff, and community resources; (3) think critically for evidence-based practice; (4) implement school health services and programs; (5) integrate legal and ethical nursing practice; and (6) conduct health education.

## 4. Discussion

This study developed a school nurse competency framework for continuing education for Korean school nurses. The present study defined school nurse competencies as the knowledge, skills, and attitudes required of school nurses to provide safe school healthcare. This definition supports a concept analysis of competency in nursing [20], namely, a framework for continuing education emphasizing the mastery of competency in the domains of knowledge, skills, and abilities [9]. The present study identified six core competencies, similar to those presented in the papers on a well-known framework [5], practice standards [16], and roles of school nurses [4,7].

The first core competency of school nurses was the ability to provide patient-centered care through the integration of knowledge and skills, which others also have identified as an essential competency of school nurses [4,5]. School nurses should be able to respond appropriately to a variety of health needs of students and staff in schools. Asthma, attention deficit hyperactivity disorder, and severe allergies were the most common health conditions in schools between 2006 and 2016 [21]. However, the outcomes of school nurse-led practice regarding mental health [22] and pain have not been satisfactory [23]. Furthermore, most school nurses have had no formal training in evidence-based practice [24]. Therefore, it is necessary to design and implement a continuing education program that can help school nurses carry out evidence-based nursing practice.

The second core competency was the ability to communicate and collaborate with students, school staff, and community resources, which previous studies also have identified as an essential competency of school nurses [1,5,15]. School nurses should actively collaborate with parents, teachers, medical advisors, and stakeholders to increase the student and family capacity for adaptation and self-management [7]. One review also identified motivational interviewing and counseling as an important competency in the majority of school nurse interventions and activities [2]. Seen in one study, a school nurse-led intervention highlighting collaborative communication between school nurses and parents improved healthy behavior [25]. The communication and collaboration competencies are important for positive outcomes in school nurse-led interventions and activities, necessitating competency-building programs for school nurses.

The third core competency was the ability to think critically. An empirical referent of critical thinking is the clinical competence of offering protection and safety among nurses serving populations [26]. The results from our focus group interviews reveal that the most preferred theme among the school nurses was basic life support, followed by first aid, safety, and accidents. The Council on School Health [4] also emphasized the role of school nurses in emergency preparedness. Since various emergency health problems can occur in schools, school nurses should act as care coordinators. Therefore, scenario-based continuing education is needed for school nurses to acquire and apply critical thinking skills effectively.

The fourth core competency was the ability to implement school health services and programs. The school nurse plays a vital role in timely disease management, as well as public health services [4]. However, one survey identified a gap between the importance and performance for quality improvement and community/public health practice [27]. Best et al. [21] mentioned noticeable increases in health services for asthma, type I diabetes, healthcare procedures, school nurse-led health counseling, and diabetes health counseling. There are more children and adolescents with chronic and special public health problems now than in the past. This scenario warrants nurse educators to design a competency-based continuing education curriculum that enables school nurses to design, implement, and evaluate school health services considering the changes in the healthcare environment.

The fifth core competency was the ability to integrate legal and ethical nursing practice. Laws and policies were regarded as self-identified barriers to the nurses’ ability to practice school nursing [27]. There is an increasing demand for school nurses to provide continuing education regarding specific education laws [16]. Currently, child abuse and school violence are legal and ethical school health issues in Korea, so school nurses must be able to respond appropriately. School nurses face student healthcare legal mandates, necessitating the provision of a continuing education curriculum so school nurses, as advocates, can follow the proper legal and ethical principles.

The sixth core competency was the ability to conduct health education, one of the important domains of the school nurses’ role to achieve primary prevention [10,16]. School nurses should design and deliver health education for students, families, school staff, and the community to achieve optimal levels of wellness [28]. One study identified the priority health education topics as hand hygiene, tooth decay and oral health, sexual health, smoking, mental health, obesity, and healthy eating [7]. According to the FGIs in the present study, school nurses wanted to reflect the current updated domestic and foreign health issues in selecting the topics for health education. However, school nurses face difficulties in finding adequate educational resources, finances to access resources, and human resources to deliver health education [7]. There is a need for a continuing education program to design and implement health education, considering the high health priorities of the students and the contextual issues regarding health education.

The FGIs in the present study demonstrated that the school nurses’ most preferred themes of continuing education are basic life support, followed by first aid, safety, and accidents. Emergency preparedness is one of the important roles of school nurses in providing school health services [4]. The number of school safety accidents in the Republic of Korea increased from 6947 in 2008 to 12,570 in 2018, a 56.6% increase in 10 years [29]. Therefore, nurse educators should provide continuing education to improve the school nurses’ emergency preparedness competency.

The present study also identified the demand for continuing education, depending on the school nurses’ expertise. The school nurses’ work experience, clinical experience, and training are important factors in implementing school nursing [30]. Regarding Korea, the clinical career stage for the completion of continuing education for school nurses is not classified. Based on the framework developed in the present study, nurse educators need to identify the highest priority competency domains to be strengthened through continuing education and present the appropriate continuing education according to the expertise level of the school nurses. The significance of this study is as follows. Since school nurses play an essential role in school healthcare, nurse educators need to design and implement continuing education programs to develop and maintain school nurse competency. The present study suggested a school nurse competency framework for continuing education that could be adopted by nurse educators for developing a continuing education program for school nurses. 

The limitations of this study are as follows. First, because only the school nurses from 11 school districts in a capital city of the Republic of Korea participated in this FGI, the findings cannot be generalized to the school nurses of other countries. Second, because the present study only verified the content validity by the expert group on the competency domains and its indicators, it is necessary to additionally confirm the reliability and validity of the developed school nurse competency indicators.

## 5. Conclusions

School nurses play an important role in the school setting and are responsible for the health of students with diverse healthcare needs. Therefore, it is important to design and implement effective continuing education to ensure the school nurses’ optimal competency. However, the research on such a school nurse competency framework for continuing education in Korea is lacking. The present study identifies six core competencies of school nurses: patient-centered care, communication and collaboration, critical thinking ability, school health services, legal and ethical nursing practice, and health education. The competency of the school nurses can influence the health outcomes of the school members, so an effective strategy is needed to develop and improve the school nurses’ competency. The current continuing education system for school nurses in the Republic of Korea has not changed much since 2015 when this focus group interviewwas conducted. Therefore, nurse educators could extend the study results to the framework of the continuing education for school nurses. Further study is required to verify the short- and long-term effects of competency-based continuing education for school nurses.

## Figures and Tables

**Table 1:** Preferred Themes of Continuing Education.

Domains	Themes	Subthemes
Assessment and treatment	Training regarding assessment and treatment of common diseases	Anatomy and physiology Assessment of pain (namely, distinction of stomachache, headache, chest pain, hip joint pain, arthralgia and growing pain, etc.) and its treatment Assessment and treatment of each fracture type Judgment of emergency when children request help Ability to screen genuinely sick students
	Training regarding assessment and treatment of severe diseases	Use of defibrillator in cardiopulmonary resuscitation Assessment and management of students with syncope, unconsciousness, altered mental status, and airway obstruction Infectious diseases Student cases with various health problems
	Training regarding assessment and treatment of rare diseases	Musculoskeletal problems (spine dichotomy, osteoporosis) Emergency treatment of chronic diseases like diabetes mellitus Common gender-specific diseases
Situation management	Dispute resolution	Preventing legal disputes Responding to parent claims
	Managing school violence	Emergency treatment for school violence Suicide attempts Coping with self-harm situations
Task management	Managing the school health room	Use of health equipment and medical devices General medicine and new medicine
